# Retinal Axon Interplay for Binocular Mapping

**DOI:** 10.3389/fncir.2021.679440

**Published:** 2021-06-04

**Authors:** Coralie Fassier, Xavier Nicol

**Affiliations:** Institut de la Vision, Sorbonne Université, INSERM, CNRS, Paris, France

**Keywords:** cAMP, retina, dorso-lateral geniculate nucleus, retinal ganglion cells, axon, binocular map, competition, cooperation

## Abstract

In most mammals, retinal ganglion cell axons from each retina project to both sides of the brain. The segregation of ipsi and contralateral projections into eye-specific territories in their main brain targets—the dorsolateral geniculate nucleus and the superior colliculus—is critical for the processing of visual information. The investigation of the developmental mechanisms contributing to the wiring of this binocular map in mammals identified competitive mechanisms between axons from each retina while interactions between axons from the same eye were challenging to explore. Studies in vertebrates lacking ipsilateral retinal projections demonstrated that competitive mechanisms also exist between axons from the same eye. The development of a genetic approach enabling the differential manipulation and labeling of neighboring retinal ganglion cells in a single mouse retina revealed that binocular map development does not only rely on axon competition but also involves a cooperative interplay between axons to stabilize their terminal branches. These recent insights into the developmental mechanisms shaping retinal axon connectivity in the brain will be discussed here.

## Introduction

**Figure 1:**
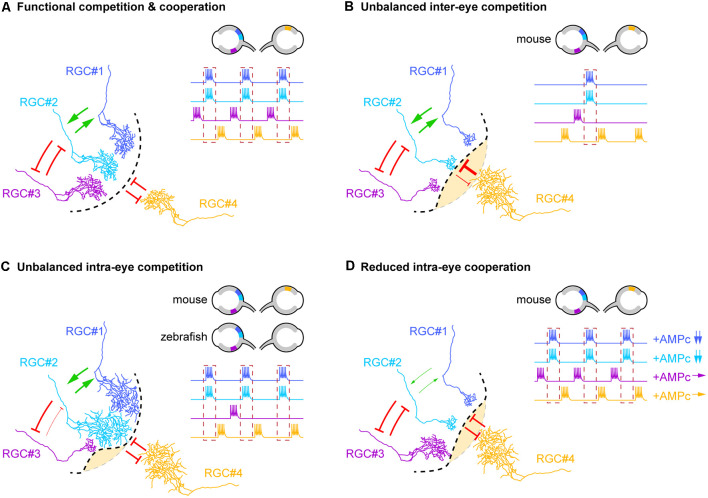
Competitive and cooperative axonal interplay shape binocular maps. **(A)** Diagram illustrating the different types of inter- and intra-eye axonal interactions that contribute to the segregation of eye-specific territories in the dorsolateral geniculate nucleus (dLGN) and superior colliculus (SC). Competitive interactions (red arrows) between retinal ganglion cell (RGC) neurons from opposite eyes (RGC#1, #2 and #3 vs. RGC#4), non-correlated neurons from the same retina (RGC#2 and #3) as well as cooperative interactions (green arrows) between correlated neighboring RGCs from the same eye (RGC#1 and RGC#2) drives the refinement of terminal arbors underlying binocular mapping. The dotted black line delineates the frontier between eye-specific territories. The dotted red box on the RGC firing profiles highlights neurons that fire in synchrony (RGC#1 and #2). Retinal localizations and firing profiles of RGCs involved in these interactions are schematized in the top right corner. **(B–D)** Schematic representation of the anatomical consequences associated with the dysfunction of each type of axonal interactions. The changes in cooperative or competitive interplay reflected on the schematics are restricted to the interactions directly modified by the experimental manipulations. The dotted gray line indicates the position of the frontier that separates each eye territory under physiological conditions. **(B)** Unbalanced inter-eye axon competition induced by genetic, pharmacological, or optogenetic modulations of neuronal activity in mouse pups leads to the enlargement of the territory of the more active retina at the expense of the territory of the opposite eye (yellow area). **(C)** Similarly, unbalanced intra-eye competition obtained through the genetic reduction of axonal density in the SC/tectum of mouse/zebrafish embryos induces an exuberant growth of the remaining retinal arbors from the same retina. In mouse pups, this phenotype is also associated with an expansion of the territory occupied by RGC axons from the opposite eye. The reduction of neuronal activity or evoked synaptic release in a single RGC of zebrafish larvae leads to the shrinkage of the terminal arbor of this axon. **(D)** Reduced intra-eye cooperation between correlated neighboring RGCs induced by the reduction of cAMP signaling (in RGC#1 and #2) impacts the costabilization of axonal branches, thereby reducing their terminal arbor size and allowing the expansion of terminal arbors from the opposite eye.

**Figure 2:**
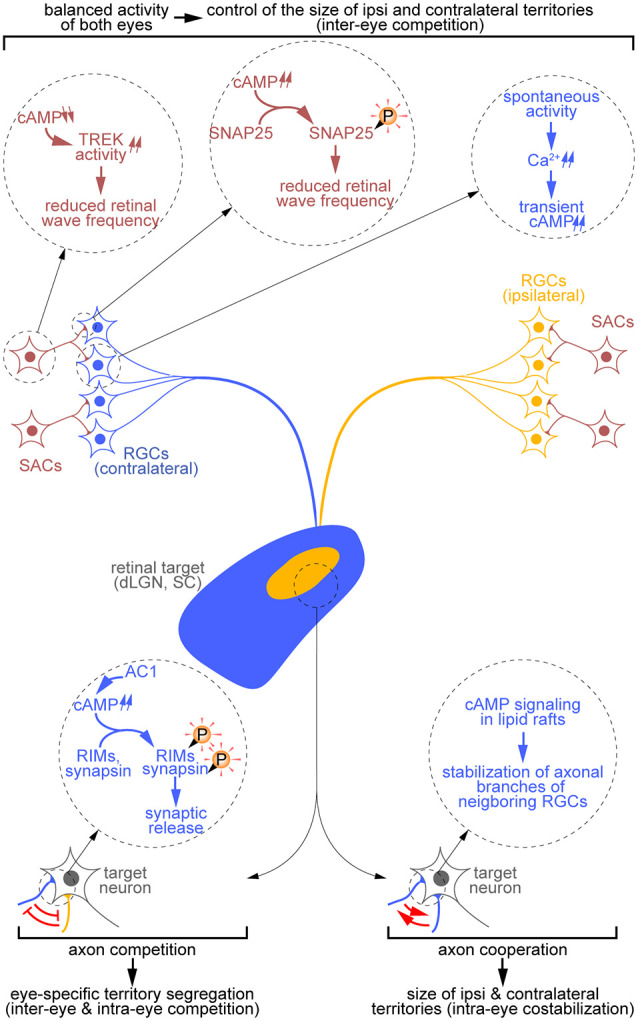
Diversity of cAMP-dependent mechanisms controlling the development of eye-specific territories. cAMP signals influence the remodeling of RGC terminal arbors both indirectly through the generation of retinal waves (top) and directly through the regulation of RGC axon branch dynamics and synaptic release (bottom). In starburst amacrine cells (SACs; red), a reduction of cAMP concentration controls the activity of the TREK channels, thus influencing the refractory period of these cells. By contrast, at the SAC-RGC synapse, cAMP signaling elevation drives the phosphorylation of SNAP25 and reduces the frequency of retinal waves. Spontaneous activity-driven cAMP transients are also detected in RGCs (blue, contralateral; orange, ipsilateral). Extending the observations made at the somatosensory thalamocortical synapse, one can hypothesize that axon competition requires cAMP-dependent regulation of synaptic release at the retinothalamic synapse (bottom left). Finally, retinal axon cooperation relies on cAMP signals restricted to lipid rafts (bottom right).

The accurate processing of visual information relies on the precise tuning of visual system connectivity. In most mammals, including rodents and humans, each hemisphere of the brain receives afferences from retinal ganglion cells (RGCs) of the same (ipsilateral) and opposite (contralateral) side of the body (Godement et al., 1984). In mature organisms, terminal arbors of ipsi and contralateral RGC axons are organized in non-overlapping territories in their main brain targets—the dorsolateral geniculate nucleus (dLGN) and the superior colliculus (SC)—enabling binocular vision. At birth, the terminal arbors of ipsi and contralateral axons are exuberant in the dLGN and SC and form overlapping areas. These exuberant projections are subsequently pruned during the two first post-natal weeks in mice, leading to the segregation of ipsi and contralateral axons into eye-specific territories. The refinement of RGC terminal arbors requires waves of spontaneous activity that propagate throughout the retina (Shatz and Stryker, 1988). Notably, whereas correlated electrical activity within a retina is a prerequisite for the development of eye-specific territories, non-correlated activity between both eyes is critical for the development of binocular maps (Zhang et al., 2012). In addition, the relative level of activity between both retinas controls the extent of the territory occupied by the axons from each eye, leading to the idea that competitive interplay between retinal axons from opposite eyes contributes to the shaping of binocular maps (Penn et al., 1998). Such inter-eye competitive mechanisms have been thoroughly investigated. By contrast, the influence of interactions between mammalian RGC axons from the same retina has been scarcely addressed so far. We here review the mechanisms underlying retinal axon interactions required for binocular mapping (Figure 1). We provide a brief overview of the inter-eye competitive mechanisms (reviewed in Assali et al., 2014) and further describe the studies that explored the interplay between axons from the same eye (intra-eye interactions), including those conducted in vertebrate species lacking ipsilateral RGCs. Finally, since both inter and intra-eye retinal axon interactions require cAMP signaling, we summarize the divergence between the cAMP-dependent mechanisms involved in inter-eye axonal competition and intra-eye cooperative interplay (Figure 2).

## Competitive Interplay Between RGC Axons Shapes Binocular Maps

### Inter-eye Competitive Interactions in the Segregation of Eye-Specific Territories

Competitive interactions between RGC axons from opposite retinas were identified early as a key mechanism in binocular map formation (Figures 1A,B; Penn et al., 1998) and were postulated to involve Hebbian synaptic learning rules, which are based on correlated afferent activities (Mu and Poo, 2006; Butts et al., 2007). In some mammalian species including rodents, binocular map formation occurs before eye-opening and thus before visual input (Wong, 1999). However, the existence of waves of spontaneous activity that propagate throughout the developing retina compensates for the lack of visual experience. Retinal waves are characterized by specific spatiotemporal properties and synchronize the activity of neighboring RGCs (Feller et al., 1996; Bansal et al., 2000). Over the past decades, a combination of pharmacological, genetic, and optogenetic approaches have been used to dissect the role of retinal waves in binocular mapping. Monocular elevation of retinal wave activity (e.g., by increasing cAMP levels) induces the expansion of the territory occupied by RGC axons from the more active retina in the dLGN, at the expense of the area covered by afferences from the less active retina. Such a phenotype is not observed following binocular stimulation of retinal activity, demonstrating that activity-based competitive interplay between retinal axons from each eye drives the segregation of eye-specific territories (Stellwagen and Shatz, 2002). However, whether retinal waves play a permissive (i.e., a minimum level of activity is required for binocular mapping) or instructive (i.e., spatiotemporal properties of retinal waves influence binocular mapping) role in the formation of binocular maps remained to be clarified. This controversy was elegantly solved by studying transgenic mice in which spontaneous cholinergic waves were spatially reduced without affecting the overall activity of RGCs (Xu et al., 2011). This slight alteration of the structural properties of spontaneous activity is sufficient to impair the segregation of eye-specific territories in the SC and dLGN, suggesting that retinal activity is not sufficient to shape binocular maps and that structured and correlated activity between neighboring RGC axons is required to fine-tune visual map connectivity (Xu et al., 2011). Consistently, *in vivo* synchronous or asynchronous optogenetic stimulation of both eyes reduces or enhances the segregation of axons from opposite retinas into eye-specific territories, respectively (Zhang et al., 2012). This study demonstrates that the temporal features of retinal waves are critical for inter-eye competitive interactions underlying binocular map refinement. The competitive interplay between RGC axons from both eyes was further shown to rely on synaptic transmission (Assali et al., 2017). Notably, the inhibition of glutamate synaptic release in ipsilateral axons affects the segregation of eye-specific territories in the dLGN, while preserving their size. These results identify glutamatergic transmission as an additional key mechanism in the inter-eye competition-driven refinement of binocular maps (Koch et al., 2011).

### Inter-eye Competitive Interactions in the Dynamic Remodeling of RGC Terminal Arbors

*In vivo* investigations in mammals have provided important insights into the regulation of binocular map refinement at a structural/anatomical scale (e.g., position, size, and overlap of the territories occupied by retinal axons from each eye). However, *in vivo* dynamic analyses at the cellular scale are essential to provide mechanistic clues regarding the influence of patterned neuronal activity on retinal axon branch dynamics (i.e., branch growth, addition, stabilization/elimination). Tackling this issue is challenging in mice but accessible in other optically transparent vertebrates such as albino *Xenopus* tadpoles. While retinotectal projections are entirely crossed in *Xenopus*, unilateral ablation of the tectum (equivalent to the SC in mammals) drives the axonal rewiring of RGCs contralateral to the ablation site. These axons thus innervate the remaining ipsilateral tectum, which they would not normally target. This surgical approach generates an artificial binocular system compatible with the *in vivo* monitoring of branch dynamics underlying inter-eye axonal competition. Using this experimental system, Rhuthazer et al. demonstrated that RGC axons from both eyes have the same probability to form new branches in all tectal territories. However, retinal axons preferentially drive branch elimination in territories dominated by the opposite eye, while stabilizing new branches in appropriate territories, two NMDA receptor-dependent processes (Ruthazer et al., 2003). The instructive role of correlated activity in synaptic maintenance and axonal refinement has further been described by Muntz et al. who exploited the few *Xenopus* tadpoles exhibiting a single misguided axon projecting to the ipsilateral tectum. These animals enabled them to assess how synchronous or asynchronous stimulation of this lone ipsilateral axon relative to its axonal neighbors influences arbor dynamics and drives tectal post-synaptic partners. Non-correlated RGC axons rapidly lose their ability to trigger an action potential in their postsynaptic partners and add new exploratory branches to their terminal arbor. Conversely, correlated retinal axons form fewer novel branches, but these branches are more stable and maintain their synaptic contacts. This dynamic behavior of synchronized axons relies on NMDA receptors, suggesting a correlation-based Hebbian remodeling of binocular mapping (Munz et al., 2014). On the other side, the exploratory axon branching behavior of neighboring RGC axons that fire out of synchrony was recently suggested to rely on a non-cell-autonomous Stentian signal that induces the loss of synaptic contacts of non-correlated neighboring axonal inputs (i.e., the opposite of a Hebbian signal; Rahman et al., 2020). This hypothesis is based on the observation that the addition of new branches in the ipsilateral axon is exclusively increased by contralateral eye stimulation and is enhanced by tetanus toxin-mediated silencing of the ipsilateral axon.

Altogether, investigations in tadpoles highlight that Hebbian and Stentian mechanisms control the dynamic remodeling of axonal branches, thus influencing the activity-based competitive interplay required for the segregation of retinal axons in eye-specific territories.

## Interplay Between Retinal Axons from The Same Retina in Retinal Arbor Remodeling

### Competitive Interactions: Lessons From Model Systems Lacking Binocular Maps

Studies in mammals and tadpoles revealed that the fundamental aspects of visual map development are conserved among vertebrates, including the refinement of terminal arbors by inter-eye activity-based competitive mechanisms. However, studies from the zebrafish model, which completely lacks ipsilateral projections, revealed that competition between RGC axons from the same eye is also critical for RGC terminal arbor remodeling (Kita et al., 2015). By transplanting a few blastomeres from zebrafish embryos expressing GFP in RGCs in a mutant lacking RGCs, Gosse and collaborators created zebrafish chimeras with eyes containing a single RGC. Using this model, they showed that axonal density in the tectum directly influences the refinement of terminal arbors of RGC axons from the same eye. They further demonstrated that intra-eye competitive interplay between RGCs does not contribute to the initial positioning of their terminal arbors but is required to fine-tune their final arbor size and shape (Gosse et al., 2008). Strengthening this idea, zebrafish mutants with altered neuronal activity exhibit defects in retinal arbor size (Trowe et al., 1996; Gnuegge et al., 2001; Smear et al., 2007). Notably, in the vGLUT2 (vesicular glutamate transporter 2) *null* mutant, the exuberance of retinal arbors associated with the lack of presynaptic glutamate release increases the receptive field of tectal neurons and impairs visually-driven behaviors (Smear et al., 2007). These data demonstrate that intra-eye axonal competition requires glutamatergic transmission, like inter-eye axonal competitive interplay in mice (Koch et al., 2011). In addition, the zebrafish optical transparency and straightforward genetics have been major assets to clarify the modalities of the competition rules operating between the same eye neighboring axons (intra-eye competition). Indeed, they allowed the real-time imaging of a single RGC terminal arbor while suppressing its activity (or synaptic transmission), in combination or not with the silencing of its neighbors. Using these approaches, two independent groups consistently revealed that activity-dependent intra-eye competition controls retinal axon arbor refinement. However, both groups reported contrasting effects of activity loss on branch growth dynamics, which can partially be explained by the different tools used to silence neuronal activity. Indeed, reducing stimulus-evoked synaptic transmission or electrical activity decreases the size of axonal arbors, whereas the complete blockade of both stimulus-evoked and spontaneous synaptic release favors the growth of axonal branches (Hua et al., 2005; Ben Fredj et al., 2010).

Altogether, experiments conducted in zebrafish revealed the existence of a competitive interplay between axons from the same retina, which may operate through similar mechanisms as inter-eye axonal competition (Figures 1A,C). Importantly, competitive interactions between RGC axons from the same eye may also exist in rodents. Indeed, Maiorano et al., showed that the genetic loss of nasal and temporal but not central RGCs in the mouse embryo induces the remaining contralateral axons to expand in axon-depleted areas of the contralateral SC (Maiorano and Hindges, 2013). Of note, some ipsilateral axons were also abnormally distributed in these contralateral territories (*i.e.*, targeting of the anterior and posterior superficial SC from which they are normally excluded), revealing a contribution of intra-eye axonal competitive interplay in binocular mapping (Figures 1A,C), in addition to the regulation of retinotopic mapping (for review see Arroyo and Feller, 2016).

### Cooperative Interactions Between Neighboring RGC Axons From the Same Retina

While intra-eye competitive interactions contribute to the refinement of retinal arbors, the cooperative interplay between neighboring coactive RGCs was suggested to stabilize their synaptic contacts and to counteract competitive mechanisms (Arroyo and Feller, 2016). However, this hypothesis was mainly based on theoretical models, while *in vivo* experimental evidence supporting this concept was still lacking (Godfrey and Swindale, 2014). To address this issue, Louail and colleagues recently developed an approach enabling the differential manipulation and labeling of neighboring RGCs of a single mouse retina. Using this strategy, they showed that reducing cAMP signaling in a limited number of RGC axons is sufficient to diminish the density of neighboring retinal axon arbors with intact cAMP signaling in the contralateral dLGN. This phenotype emerges between P3 and P15, suggesting that cooperative and competitive mechanisms occur at the same developmental stages. These results highlight that neighboring axons cooperate to costabilize their terminal arbors in the dLGN *via* a non-cell autonomous mechanism that requires cAMP signals. They further demonstrate that altering cAMP signaling in a subset of contralateral RGC axons induces an enlargement of the territory occupied by ipsilateral axons from the non-electroporated eye. This suggests that cAMP-dependent cooperative interplay between neighboring RGC axons is also required for balanced binocular competition (Figures 1A,D; Louail et al., 2020).

In conclusion, this study provides the first *in vivo* evidence for a cooperative interplay between neighboring RGCs that is required for retinal axon terminal arbor shaping and binocular map development.

## cAMP-Dependent Mechanisms Underlying Axon Interplay

As mentioned above, cAMP signaling influences several mechanisms that regulate both competitive and cooperative interactions between axons. Strikingly, distinct cAMP manipulations differentially impact the development of retinal maps. For example, elevating cAMP concentration in one retina enlarges the area covered by the axons from this eye, but does not alter the segregation of binocular territories (Penn et al., 1998). By contrast, the deletion of one of the cAMP-synthesizing enzyme genes, adenylyl cycalse 1 (AC1), in mice leads to overlapping ipsi and contralateral areas in the dLGN and the SC (Ravary et al., 2003). Interestingly, cAMP signals located in a specific subcellular compartment of the plasma membrane, the lipid rafts, are required for the costabilization of axonal branches between neighboring RGCs (Louail et al., 2020). The different features between the cAMP signals involved in these axonal phenotypes enable us to draw hypotheses that link each cAMP signal to a specific cellular process influencing either competitive or cooperative mechanisms (Figure 2).

### cAMP-Dependent Control of Retinal Waves and Binocular Competition

The frequency and structure of spontaneous activity in the developing retina are critical determinants of RGC axon behavior during competitive mechanisms (Penn et al., 1998). These waves involve two neuronal subtypes in the retina: starburst amacrine cells (SACs) and RGCs. SACs initiate the spread of correlated activity in RGCs. Interestingly, the pan-retinal pharmacological elevation of cAMP levels increases both the frequency and the spread of retinal waves (Stellwagen et al., 1999). Consistently, monocular elevation of cAMP levels leads to the enlargement of the territory occupied by axons from the manipulated retina at the expense of the area innervated by axons from the opposite eye, without preventing axon segregation in eye-specific territories (Stellwagen and Shatz, 2002). However, the cell type and molecular mechanisms involved in this cAMP-dependent inter-eye competition remain unclear.

To address this issue, two groups achieved SAC-specific manipulations of cAMP signaling. Hsiao and colleagues demonstrated that overexpressing the presynaptic protein SNAP25, a target of the cAMP-dependent protein kinase A (PKA), reduces calcium wave frequency. This impact on retinal spontaneous activity is absent when a PKA-phosphodeficient mutant of SNAP25 is overexpressed. Their results suggest that PKA is required in SACs to decrease retinal spontaneous activity (Hsiao et al., 2019). Ford et al. used a different approach based on the pharmacological inhibition of the SAC-specific phosphodiesterase 1C to prevent the calcium-induced decrease of cAMP levels. They showed that increasing cAMP signaling induces TREK potassium channel-dependent long–lasting hyperpolarization periods in SACs, thus influencing both the refractory period of these neurons and the frequency of spontaneous bursts of electrical activity (Ford et al., 2013). These contrasting experiments might be reunified if one hypothesizes that the reduction of cAMP concentration induces a TREK-dependent reduction of SAC electrical activity, whereas the PKA-induced phosphorylation of SNAP25 in the axon terminals acts as a negative regulator of synaptic release in SACs and thus of RGC activity.

In addition to these SAC-specific mechanisms, cAMP transients synchronized with waves of electrical and calcium activity were detected in RGCs, suggesting that a cAMP-dependent mechanism restricted to RGCs may also influence spontaneous activity (Dunn et al., 2006). Altogether, three independent and sometimes conflicting cAMP-dependent pathways are associated with the generation of waves of activity in the retina (Figure 2).

### Regulation of Inter-eye Competition by cAMP at RGC Axon Terminals

To identify the source of the cAMP signals that modulate the patterns of retinal waves, spontaneous activity and calcium concentrations have been recorded in the developing retina of AC1 knockout mice (AC1^−/−^). AC1 is the only adenylyl cyclase identified to influence the development of binocular maps (Ravary et al., 2003; Nicol et al., 2006a). Surprisingly enough, the lack of this enzyme does not impact retinal waves (Dhande et al., 2012). Furthermore, cAMP transients and PKA activity are also unaltered in developing RGCs of AC1^−/−^ mice (Dunn et al., 2009), suggesting that the AC1-dependent mechanisms that influence binocular maps are distinct from the cAMP-dependent mechanisms that control retinal waves. Supporting this idea, the phenotype of AC1^−/−^ mice differs from the one associated with the overall elevation or reduction of cAMP concentration in the retina. Whereas AC1^−/−^ mice exhibit overlapping ipsi and contralateral territories in both the dLGN and SC, modulating cAMP concentration in both retinas does not impact binocular mapping. Further investigations of the AC1-dependent regulation of visual maps—using retina-specific conditional mutants or retinocollicular coculture systems mixing retinal and collicular explants from different AC1 genotypes—identified RGCs as the cell type involved, ruling out a contribution of AC1 in their postsynaptic targets (Nicol et al., 2006b; Dhande et al., 2012).

While the molecular mechanisms involving AC1 and shaping eye-specific territories have not been explored, molecular clues have emerged from studies investigating the role of AC1 in the refinement of somatosensory thalamocortical projections. Thalamic- or cortex-specific AC1 conditional knockout mice revealed that this cAMP-synthetizing enzyme is required in presynaptic neurons to control axonal branch remodeling in the somatosensory system like in retinal projections (Suzuki et al., 2015). In thalamocortical axons, AC1 regulates neurotransmitter release through the phosphorylation of the active zone proteins RIMs and synapsin (Lu et al., 2006). The developmental similarities between the visual and somatosensory systems suggest that AC1 might also control the neurotransmitter release machinery in RGCs to influence retinal axon pruning and thereby the refinement of binocular maps (Figure 2).

### Regulation of Intra-eye Cooperation by cAMP at Axon Terminals

The cAMP-dependent pathways mentioned above have been identified in studies focusing on inter-eye competition. However, intra-eye cooperative interactions, which contribute to the costabilization of terminal arbors of neighboring RGCs, also rely on a cAMP-dependent mechanism. The latter has been identified by preventing cAMP signaling in a subset of RGCs within a single retina using a genetically-encoded cAMP scavenger while labeling these RGCs differentially from their neighbors with intact cAMP signaling (Louail et al., 2020). Importantly, this scavenger was targeted to lipid rafts, a subcellular compartment of the plasma membrane where AC1 is localized in RGCs (Averaimo et al., 2016), suggesting the involvement of this adenylyl cyclase in both inter-eye and intra-eye retinal axon interplay (Figure 2). This hypothesis is supported by the observation that the cAMP-dependent mechanism driving intra-eye cooperative interactions does not influence the generation of spontaneous waves of electrical activity (Louail et al., 2020). This is reminiscent of the AC1^−/−^ mice phenotype that associates alterations in binocular mapping to unaffected retinal waves (Dhande et al., 2012). This lack of impact of RGC-specific modulation of cAMP levels on spontaneous activity further suggests that the cAMP signal stabilizing axonal arbors of neighboring RGCs might influence retinothalamic synaptic transmission. In this view, one could hypothesize that: (i) both the cAMP-dependent regulation of competitive and cooperative processes might involve the control of the neurotransmitter release machinery (see “Regulation of Inter-eye Competition by cAMP at RGC Axon Terminals” section) and that (ii) the switch from competition to cooperation would be elicited by the synchrony of the neurotransmitter release in neighboring axon terminals. This view draws a model in which a single presynaptic and cAMP-dependent mechanism would control both Hebbian and Stentian signals identified in RGCs. However, further investigations will be required to validate this model.

## Conclusion

The development of binocular maps is governed by balanced competitive and cooperative mechanisms that refine initially exuberant ipsi and contralateral territories. These mechanisms are dependent on spontaneous activity in the retina and a complex interplay between axonal branches from the same and opposite retina. cAMP signaling is involved in many steps of these cellular processes. While the overall mechanisms influencing both the generation of waves of activity in the developing retina and the interplay between axonal branches of RGCs from both eyes are mostly understood, the description of the competitive and cooperative intra-eye interactions and the molecular mechanisms governing all steps of binocular mapping will benefit from further investigations.

## Author Contributions

CF and XN wrote the manuscript. All authors contributed to the article and approved the submitted version.

## Conflict of Interest

The authors declare that the research was conducted in the absence of any commercial or financial relationships that could be construed as a potential conflict of interest.
